# Enhancing Parents’ Well-Being after Preterm Birth—A Qualitative Evaluation of the “Transition to Home” Model of Care

**DOI:** 10.3390/ijerph19074309

**Published:** 2022-04-04

**Authors:** Natascha Schuetz Haemmerli, Liliane Stoffel, Kai-Uwe Schmitt, Jeannine Khan, Tilman Humpl, Mathias Nelle, Eva Cignacco

**Affiliations:** 1 Department of Health Professions, Bern University of Applied Sciences, 3008 Bern, Switzerland; kai-uwe.schmitt@bfh.ch (K.-U.S.); eva.cignacco@bfh.ch (E.C.); 2 Department of Paediatrics, Inselspital, Bern University Hospital, University of Bern, 3010 Bern, Switzerland; liliane.stoffelzuercher@insel.ch; 3 Insel Gruppe, Bern University Hospital, 3010 Bern, Switzerland; 4 Kantonale Schule für Berufsbildung, 5001 Aarau, Switzerland; jeannine.khan@berufsbildung.ag; 5 Tilman Humpl, Department of Paediatrics, St. Elisabethen-Krankenhaus, Kliniken des Landeskreises Lörrach, 79539 Lörrach, Germany; humpl@icloud.com; 6 Mathias Nelle, Children’s Hospital, Kreiskliniken Böblingen, 71302 Böblingen, Germany; m.nelle@bluewin.ch

**Keywords:** preterm infant, parents, transitional care, early intervention, home visiting, qualitative research

## Abstract

There are few programs available aimed at preventing short- and long-term negative consequences after preterm birth and covering the entire care continuum. The “Transition to Home (TtH)” model is such a program, offering structured, individual support for families with preterm infants before and after hospital discharge. This study gathers and examines the parents’ views of receiving support from an interprofessional team under the TtH model of care during hospitalization and after discharge. Using a qualitative explorative design, 39 semi-structured interviews with parents were analyzed thematically. From this analysis, three main themes were identified: (1) TtH and the relevance of continuity of care; (2) Enhancement of parents’ autonomy and self-confidence; (3) Perception of interprofessional collaboration. Within these themes, the most relevant aspects identified were continuity of care and the appointment of a designated health care professional to anchor the entire care continuum. Emotional support complemented by non-medical approaches, along with strength-based and family resource-oriented communication, also emerged as key aspects. Continuous, family-centered care and well-organized interprofessional collaboration promote the well-being of the family after a premature birth. If the aspects identified in this study are applied, the transition from hospital to home will be smoothened for the benefit of affected families.

## 1. Introduction

Parents and their home environment have a significant influence on the development of preterm infants [1]. Parents suffer from increased stress during the hospital stay in the neonatal intensive care unit (NICU) and again during the transition from hospital to home and in the first months at home [2]. Persistent, ongoing distress can lead to disturbances in parents’ mental health, such as depression and anxiety [3,4,5]. When parents are suffering from such mental health challenges, they may be less competent in parenting and interacting with their child [1]. This, in turn, negatively influences their child’s development. In contrast, responsive parenting is associated with improved developmental outcomes and increased resilience in the child [6]. Consequently, health care provision should be aware of the potential burdens and vulnerabilities of preterm children and their parents in order to be able to support positive development and to intervene at an early stage to prevent short- and long-term negative effects [7]. To achieve this, different approaches are proposed.

### 1.1. Models of Care to Provide Family Support

The concept of family-centered care (FCC) is implemented in various health care settings. Although FCC is a general approach rather than a specific model of care, it highlights that family members must be involved in care to address patient needs. The development of a partnership between the family and health care professionals is a key feature of this concept [8]. FCC practice in an NICU can help parents to cope with anxiety, to adapt into their new role and to learn how to best interact with their infant, the effect of which is to positively influence the child’s development [9,10]. FCC interventions in an NICU empower and involve parents through partnership, help them to gain knowledge in the care of their preterm infant, address the family’s individual needs and wishes, and provide psychological support and family-oriented information and communication [8].

The concept of family-centered care is becoming widely used in the NICU. In most programs, however, parents are not fully integrated as part of the care team [11]. The Family Integrated Care (FICare) model addresses this issue. It is specifically tailored to the structures and processes in the NICU and addresses the support needs of parents [12]. The FICare model was first developed in Estonia and later adapted in Canada. The concept of FICare is based on human neonatal care and means that parents become the primary caregivers of their infants in the NICU [12]. FICare is a strengths-based FCC approach aiming at empowering parents, their learning and their shared decision making. It enhances parents’ self-efficacy after hospital discharge and improves the parent–infant relationship and the infants’ development [12]. FICare includes four main components: 1. Parent Education is a core component; 2. An education program for the entire NICU care team focusing on the importance of parents’ involvement in their infants’ care; 3. Providing an environment that welcomes parents and encourages them to be involved in their infants’ care as often as possible; 4. The active integration of parents into their infants’ care with nurses as coaches [12].

Several more specific models of care were developed with respect to positive child development, eventually including an FCC approach. Most of these programs are hospital-based intervention programs that end with the child’s discharge. Only a few programs are known that cover the entire care continuum, from birth until the fully completed transition to home. The most recently introduced or further developed programs are the Infant Behavioral Assessment Intervention Program (IBAIP) [13], the Stockholm Preterm Interaction-Based Intervention (SPIBI) [14], the transmural developmental support for very preterm infants and their parents (TOP-Program) [15] and the modified Mother Infant Transaction Program (MITP) [16]. All these intervention programs are aimed at ensuring that parents can read and understand their infant’s behaviors and are empowered to respond appropriately to the infant’s cues. This strength-based approach is founded on the idea that behavioral subsystems, such as autonomic, motor, state organization, attention, and self-regulation of the infant, interact with and are influenced by the infant’s environment [17]. There is agreement that intervention programs related to preterm infants should provide a multilevel approach [7,18]. This includes family-centered care, psychosocial support, continuity of care and interprofessional collaboration (IPC), which begins in the antepartum period, continues throughout hospitalization and helps in making the transition from hospital to home [18,19,20]. While intervention programs may start in the NICU, there is often a gap in supportive programs for families transitioning from hospital to home and after discharge. Furthermore, the evaluation of such care models is often limited. Randomized clinical trials to evaluate care models have focused on their impact on the child’s medical outcomes and development [13], while other studies have addressed the psychological impact of care programs on parents [14]. However, there is a gap with regards to evaluating such programs from the parents’ perspectives.

### 1.2. The “Transition to Home (TtH)” Model

**Figure 1:**
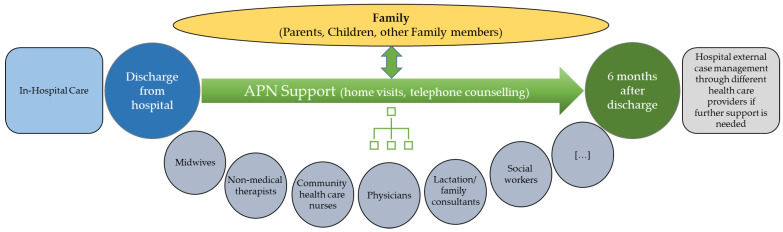
Timeline and involvement of the different professions in the “Transition to Home (TtH)” model.

In Switzerland, the “Transition to Home (TtH)” model was designed to optimize the transitional care of families with preterm infants. Focus is set on the transition from hospital to home, and the model considers the entire family in this [21]. TtH aims to give parents and infants structured, individual support in order to improve parents’ mental health and competence, to promote the child’s development and to optimize interprofessional collaboration. Table 1 summarizes the main components of the model. The TtH model includes families receiving in-hospital care and supports them until six months post-discharge (Figure 1).

As part of TtH, families are given a single point of contact. An Advanced Practice Nurse (APN), which is an academically trained nurse with a master’s degree, coordinates the interventions of the interprofessional team. Advanced practice nursing competencies include, among others, clinical practice, counselling, support of other health care professionals and leadership tasks [22,23]. While APNs are internationally established, they are still emerging in Switzerland, such that few role models are available in the context of this health care system [24,25]. In the TtH model, the APN heads a team of three nurses in the NICU.

Within the first 24 h after the preterm infant’s discharge, the APN conducts a first follow-up telephone consultation. Two further phone calls take place, approximately on day 21 and day 52. On day 3 after discharge, a first home visit takes place, followed by additional home visits approximately every 15 to 30 days. The timing and number of the follow-up calls and visits are adapted according to each family’s individual needs. In this way, the APN gets to know the families, their circumstances and their needs and provides continuous support. The APN is available during the day shift on four days (Monday to Thursday) per week.

TtH was introduced and practiced from 2018 to 2020 and is now under evaluation before it is decided whether to offer this service as part of the standard care.

### 1.3. Aims

This study aimed to explore parents’ perceptions of receiving support from an interprofessional team following the TtH model of care from birth and during hospitalization until six months after discharge. Understanding the view of the parents as key stakeholders is relevant for the development, implementation and improvement of any such model of care. Therefore, more insights are needed into how such a model impacts parents. The main research question was: “How did families experience the support and care within the TtH model?”.

Semi-structured interviews were conducted with parents to explore how the care model was perceived by them, whether they felt their well-being was adequately cared for by the interprofessional team or whether there were any aspects that were particularly important to them.

## 2. Materials and Methods

The TtH model was implemented at the University Children’s Hospital Bern, Switzerland, and was practiced between 2018 and 2020 [21]. This study was conducted as part of a comprehensive evaluation of the TtH model.

A qualitative explorative approach was used. We conducted individual semi-structured interviews at the end of the TtH support period, i.e., six months after discharge from hospital. The “Standards for Reporting Qualitative Research (SRQR)”, a 21-item checklist aimed at improving the transparency of qualitative research was used as a basis to report this study [26]. The study was approved by the responsible ethics committee of the canton of Bern, Switzerland (Project-ID: 2017–01249).

### 2.1. Participants

We chose a purposive sample of parents of preterm infants born between 24 0/7 weeks and 34 6/7 weeks of gestation, who were included in the TtH program and who followed the entire six-month support period after hospital discharge. Preterm infants were born and hospitalized in the Children’s Hospital. Parents had to reside in the canton (state) of the Children’s Hospital and speak and read German, English or French. We excluded families with preterm infants with congenital problems evident at birth. Parents who were eligible for this study were contacted by members of the research team (J.K., J.R.) after the completion of the support period and were asked to participate in an interview. A standard on-boarding process was followed, i.e., potential study participants received detailed study information and were informed about data protection procedures. All parents provided written consent to participate. Participants were coded such that all data were pseudonymized.

### 2.2. Semi-Structured Interview Guide

A semi-structured interview guide was developed (see Supplementary Materials, Table S1) by the first author together with a researcher from the neonatal field and a mother of a former preterm infant. The development of the interview guide focused on the objective of evaluating the TtH model from the perspective of the key stakeholder: the parents supported by the TtH model. The semi-structured interview guide opened with a thematically focused invitation to speak freely. Questions addressed components of the TtH model. The first part explored the experiences with the APN and the support within the TtH model in general; the second part addressed the experiences with the interventions provided by different health care professionals; the last part included questions regarding the parents’ satisfaction, feelings of self-confidence and concerns regarding their future.

We first applied the interview guide in a pre-test with four parents, and then, we adapted it thereafter, according to those parents’ feedback, to derive the final version of the interview guide. The pre-test interviews were not included in the final study sample.

### 2.3. Data Collection Procedures

The semi-structured interviews were conducted between 18 September 2018 and 23 February 2020 at the parents’ homes.

All parents who followed the TtH care model (N = 40) were eligible to participate in the interviews. We expected that a minimum number of 30 and a maximum number of 40 interviews would generate sufficiently rich data to represent patterns related to parental experience of care under TtH [27]. Mothers and fathers were interviewed separately.

The interviews were performed through four researchers (J.K., J.R., N.S., P.Z.) who were trained in interview techniques. The interviews were held in German (37 participants) and English (two participants). Interviews were audio-recorded and transcribed verbatim.

### 2.4. Data Analysis

We performed a thematic analysis (TA) with a semantic, inductive approach without using a pre-existing framework. The TA was based on the method described by Braun and Clarke [28]. It is a flexible and adaptable qualitative research method because its theoretical framework is not predefined. We used TA to identify, analyze and report patterns within data [28] and to assess the experiences, perceptions and opinions shared by our interview participants. TA allowed us to explore parents’ perceptions of the care provided within the TtH model, differing realities and specific occurrences. Three methodologically trained researchers (N.S., J.K., J.R.) conducted the analysis. One (first author, N.S.) is an expert in neonatology, and the other two are a midwife (J.R.) and an educational scientist (J.K.) with background in the research field of prematurity. The research group was supported by an expert in qualitative research methods (C.H.).

TA involves six steps to explore repeated patterns: in step one, we read and re-read the interview transcripts to familiarize ourselves with the data. To generate initial codes in step two, we inductively coded the relevant text sections and explored and identified latent themes in the data. By searching for themes, we collated codes into sub-themes in step three. In step four, we reviewed the themes. We combined sub-themes, consolidated them into themes and created a first thematic map. The research group (N.S., J.K., J.R., C.H.) engaged in an iterative process of analyzing content and generating codes and themes, which we refined in step five, defining and naming themes. The research group discussed codes, sub-themes or themes in several meetings. We aimed for a consensus about coding, sub-themes and final themes among researchers after each step. Step 6 included producing a report. ATLAS.ti.^®^ version 8, a qualitative data management program, was used to support our analysis.

We ensured rigor and trustworthiness [29] by having two researchers independently read and code each transcript separately. By discussing emerging findings, we could compare them and find a consensus on final themes; this contributed to the credibility of our results. We addressed transferability by seeking to reach data saturation. Data saturation was reached during the coding when no new information regarding the focus of this study was found in the transcripts. We thoroughly documented and described our research process and ensured confirmability by creating an electronic audit trail that included our notes and coding rationale.

## 3. Results

### 3.1. Sample Characteristics

We conducted a total of 39 interviews, 19 with fathers and 20 with mothers. One father refused to participate. All participants were couples. The characteristics of the sample are summarized in Table 2.

### 3.2. Findings

The analysis of the interviews revealed three overarching themes and several corresponding sub-themes (Table 3). The following paragraphs summarize the parents’ experiences and reflect this structure.

Generally, the parents described the care within the TtH model as a helpful, continuous and comprehensive support. However, not all parents fully understood the concept and options of the model right from the start, and uncertainties were expressed.

*“In the beginning we didn’t really know, well, what do we need to do? What (support) can we actually ask for? […] It wasn’t fully comprehensible. We didn’t know in detail what “Transition to Home” meant for us.”*

(D10; 10:18)

The parents agreed that the support was essential in complex situations, especially when the premature infant was their first child. Complex situations that were mentioned included (a) infants who were dependent on medical treatment, such as tube feeding, administration of oxygen or medication; (b) families with little social support; (c) families that were unfamiliar with the Swiss health care system and (d) multiple births.

#### 3.2.1. TtH and the Relevance of Continuity of Care

Parents emphasized the continuous care that was provided through the TtH approach; it was of utmost relevance to them. The continuity of care made the transition from hospital to home smoother and strengthened parents’ self-confidence.

*“[…] it (the in-patient care) does not end all of a sudden […], it is a somewhat stepwise leaving the hospital. In my view this is very good.”*

(D7, 7:160)

Parents judged the six-month duration of the TtH support differently. All parents agreed that intensive support was most needed during the first weeks after discharge from hospital, and they valued that the duration of the support could be adjusted to their needs. Some families no longer needed the support after a couple of weeks, while others wished for a longer support period.

*“In my view you need the support especially after discharge, then, actually, you would need it 24 h/7 days. But pretty soon thereafter you don’t need it anymore.”*

(D64; 64:14)

Parents who experienced the six-month period as appropriate or even too short were those who needed particular support regarding their infant’s nutrition.

The primary care nurse, the lactation consultant, the psychologist and the APN substantially contributed to the continuous care of the families. A primary care nurse was involved if the premature infant was hospitalized for a longer period. They took over most of the direct care of the infant and the support of the parents. The parents established a trustful relationship with the primary care nurses and described them as the key caregiver on the ward.

The parents were advised by the same lactation consultant in the NICU and at home. According to the parents, the continuous lactation counselling was consistent and built on what they had already learned. The counselling strengthened the mother’s role and confidence in breastfeeding their infant.

*“Without her (lactation consultant) I hadn’t managed the breastfeeding.”*

(D5; 5:22)

The psychologist actively contacted the parents after birth and introduced their services. It was mainly the mothers who had contact with the psychologist. Mothers were able to ask for support when they knew the psychologist and the related services. Mothers who used psychological support after hospital discharge underlined how important it was to be familiar with the psychologist from the hospital. They quickly regained trust and engaged in a coping process.

Having a defined health care professional with the APN as an anchor made parents feel empowered to cope with the preterm infant’s situation at home.

##### The Advanced Practice Nurse: The Bridge Home

In our model, the continuous care was mainly provided by the same APN in the hospital and at home. The APN’s constant involvement led to the APN understanding the specific family situation and the family’s developmental process. This ensured that the APN recognized and addressed family challenges at an early stage.

*“Well, surely, many issues could be recognized early on that otherwise had potentially caused problems.”*

(D2; 2:114)

The parents expressed that they felt stable, secure, confident and less stressed when they knew that an already familiar health care specialist from the hospital could be contacted when they were back at home.

The APN closely supported the direct transition home. During the first telephone consultation (within 24 h), the parents described their initial experiences at home. Parents were mainly concerned about nutrition, sleep and behaviour of the child. These challenges were followed up on the APN’s first home visit, on the third day after discharge. This home visit was experienced as particularly helpful by the parents.

*“When she (the APN) visited us the first time, it was just the right moment. He (the child) had cried all night and didn’t drink. I was glad to know that she (the APN) was due to visit such that I could ask her whether this behaviour was normal or not […] she then checked him […] and gave me an all-clear […] this was such a relief.”*

(D4; 4:37)

The close support during the transition to home empowered parents in taking over the caregiver role. The APN was also available to support the parents when the child was readmitted to the hospital. The presence of the APN, the familiar health care professional, provided the parents with confidence and security.

In summary, parents agreed that the APN built a stable bridge between inpatient and outpatient care through the continuous support provided during the transitions. The APN’s bridge function included the coordination of consultations, discussions and information. The coordination provided by the APN relieved the parents of a substantial burden. It was very important to parents that they had a primary contact person and that they could address their concerns directly to one person. This direct contact meant that parents did not have to repeat their history several times, and they were independent of the presence of the various professionals.

*“What was most important, in my view, was this contact person. The APN […] who we could contact […] she knew us and knew how things were…”*

(D11; 11:40)

The APN played the role of a “case manager” for the parents. The APN was able to coordinate in a goal-oriented way if she was well connected with other healthcare providers and supporting institutions in the hospital and outside the hospital. The APN’s solid networking led to an immediate, seamless flow of information between health care professionals and parents. The information flowed because the APN liaised between parents and health care professionals; this meant that parents’ issues were addressed promptly, and unnecessary discussions were avoided. Parents consequently felt well informed.

The APN played the role of a mediator. If necessary, she involved other relevant health care professionals, such as the psychologist, the social worker, nurses from the family advisory service or community health care nurses. Most parents described that they felt relieved by this. The parents felt further relieved and supported by the APN’s help with administrative tasks: she obtained records from other health care specialists, provided records or obtained prescriptions.

The APN attended the parents’ meetings with other health care professionals and mediated the situation. This mediating role was particularly important in complex situations when parents were at risk of losing the overview. In such situations, the APN kept the overview, clarified questions and translated the content of conversations that were hard for parents to understand. The parents felt confident in and well supported by this approach.

##### Continuity of Care and Parents’ Unmet Expectations

While the continuity of care was often reflected on positively, parents also experienced disruptions, such as a transfer to another ward while in hospital (e.g., from NICU to the intermediate care unit), a rehospitalization or changes among the key responsible staff. Parents felt poorly prepared for such disruptions, for example, when they experienced a transfer of their preterm infant to a different unit as sudden because they were not informed beforehand. They reported such situations as stressful, resulting in insecurity or anxiety.

*“When I came to the NICU, the bed of my child had gone.”*

(D24; 24:68)

In such disruptive situations, parents lost something they were familiar with, like a certain clinical environment or a particular member of staff. Some described this as a culture shock as they had to adapt quickly to a new situation, which often also involved a different attitude towards them or different expectations of them.

*“I think what the experts don’t understand so much is the fact that the cultures are so very different.”*

(D3; 3:119)

While, for instance, parents were not much involved in the care in the NICU, they were supposed to take responsibility for many aspects of care in the intermediate care unit. If the APN, as primary contact person, was not present in disruptive situations, such as the transition from the NICU to intermediate care, parents particularly missed the APN’s support and guidance.

*“Well, this was a huge difference to the neonatal intensive care unit. At the NICU, we basically had to ask for permission if we wanted to do something, and now on the intermediate care unit, they assumed that we’d just do and only ask if we needed something.”*

(D20; 20:17)

It was only retrospectively that some parents realized that the transfer from the NICU to the intermediate care unit was related to a positive development in their child’s health and thus a step towards discharge from hospital.

Likewise, parents experienced a frequent change in clinical staff as disruptive. Some professions, such as the physiotherapists, for example, worked in larger teams and were unable to ensure that a patient was always seen by the same therapist. Some parents thus missed the opportunity to build a relationship with the health care professional.

#### 3.2.2. Enhancement of Parents’ Autonomy and Self-Confidence

The parents agreed that the needs and the support required vary from family to family. Generally, it was important to them that they felt heard and recognized. They wanted their needs and concerns to be taken seriously by the health care team, they wanted to be regarded as partners with respect to their preterm infant’s care and they wanted to be involved in decision-making. As soon as possible, they wanted to be involved in the preterm infant’s care, such that they could learn from the health care professionals. Nurses were the primary contacts during the in-patient phase and helped to make parents feel empowered to care for their child. A cooperative relationship with the health care professionals allowed parents to acquire knowledge and competencies related to their preterm infant’s care. This strengthened their autonomy.

Most of the fathers, however, responded that the support provided through the TtH model did not sufficiently address them. Most fathers had to return to work shortly after birth and/or were busy looking after siblings of the preterm infant.

*“[…] (it was a) triangle: work, hospital, going home to sleep and again work, hospital, home to sleep […] a tight timetable also without any time for us as a couple.”*

(D50; 50:31)

Consequently, they were less involved in the care of their preterm infant, which was perceived as stressful. The fact that most conversations with health care professionals, therapies and the home visits of the APN took place during the day, when the fathers were often absent, added to this negative experience.

In counselling, it was particularly important for the parents that their concerns were listened to and addressed. The APN played a key role with an explicit family-centered approach. The APN focused on challenges and suggested practical solutions and, together with the parents, identified the interventions that were most appropriate for them. This enhanced the parents’ autonomy, competence and self-confidence.

*“She (the APN) always pointed-out options […] and left it to us to decide […] and whatever we went for, it was good. We felt very well supported.”*

(D20; 20:53)

Parents enhanced their competence and confidence also through obtaining information and acquiring knowledge; this was reported as important.

##### Acquiring Knowledge

Gaining knowledge was particularly important during the first hours and days at home, since this period was characterized by parents feeling uncertain about coping with the new situation and taking over full responsibility for their infant’s health. The alternating support of the midwife and the APN helped parents meet this responsibility with confidence.

The home visits were considered as valuable by most of the parents. Parents responded positively to the ability to consult the APN on a variety of topics related to the development of their child.

*“We noticed that she (the APN) was very well prepared for the home visits. She got back to topics that we had mentioned during our last conversation, and she had answers. She really made an effort to impart knowledge. This was a huge additional value.”*

(D25; 25:46)

The APN checked the health and development of the child on every home visit together with the parents. Receiving this expert feedback and being able to discuss their individual situation was reassuring for the parents.

*“The APN and this program helped us to see the difference in her (the child’s) development from the beginning until now. […] So, we really pay more attention to how things go.”*

(D39; 39:127)

Many parents experienced feeding as a challenging issue. The requirement that the preterm infant should gain weight was often reported as a stressful pressure.

*“Body weight is a key factor, you want to know if she (the child) is gaining weight, is drinking enough, you feed her correctly […] in this aspect she (the APN) helped.”*

(D41; 41:185)

Few parents, particularly those of preterm infants born at a higher gestational age, expressed their concern that the APN was not sufficiently competent to address their specific questions, for example, related to breastfeeding or nutrition at home. The APN had a deep knowledge related to preterm infants, but according to these parents, the APN lacked a background on healthy infants’ situations. These parents preferred to consult more closely with a midwife or the parental counsellor of the family advisory service.

*“Sometimes the consultations were not about fields in which she (the APN) was strong […] perhaps this was because of my questions, but, when you are at home, it is about the child at home, not so much about preterm birth.”*

(D4; 4:11)

Topics related to breastfeeding and nutrition were regarded as particularly important. Breastfeeding advice was described as essential by many parents in the hospital and at home; it was therefore favored over other topics. The lactation consultant was perceived as highly competent. Besides the educational aspect, mothers felt supported, motivated to breastfeed, and strengthened in their self-belief that they were capable of breastfeeding. In complex cases in which breastfeeding was, for example, not possible, the advice was helpful and reduced the pressure of being able to breastfeed that the mothers placed on themselves.

Likewise, the parents appreciated if they were involved by the physiotherapists during their stay at hospital. Receiving instructions regarding how to move or support their preterm infant and learning about exercises that they could apply at home or in daily activities was described as very valuable. The parents noted the benefit of physiotherapeutic interventions for the well-being of their infant, and they observed that they felt more confident in handling the child after being instructed.

##### Parental Emotional Support

The interviews revealed that the TtH model also contributed to the emotional well-being of the parents. Parents described situations when they felt left alone or moments of anxiety or insecurity. In such moments, the support and/or recognition by the APN and/or other clinical staff was particularly appreciated and contributed to their sense that they were able to handle the current situation.

*“Most important is this encouragement, to know that you’re doing it right.” *

(D42; 42:63)

Music therapy offered during the hospital stay as one means of addressing their emotional needs and to relax and bond with the preterm infant was very positively described by all parents. This therapeutic approach allowed parents to generate positive emotions.

*“Relaxing, taking time […] the music is supporting […] it calms down […] and it is also unconsciously knowing that nothing else will happen for the next 20 min.”*

(D50; 50:67)

Several parents subsequently used music or singing later in their daily activities to promote a sense of calm in certain situations.

As a further element of the TtH model, psychological support was offered. Generally, only a few parents made use of this support. Some mothers were in contact with a psychologist during the hospital stay and required psychological support to cope with their experiences during and after the premature birth. The support was described as competent, helpful and reassuring.

*“I saw the psychologist. She was very supportive such that I felt more confident and comfortable in my role as a mother and started to build trust in the relationship (to the child). I no longer felt anxious or guilty as at the beginning.”*

(D6; 6:82)

Fathers barely made use of this component of the care model: some commented that the support was not needed, while others preferred support outside of the TtH offer.

#### 3.2.3. Perception of Interprofessional Collaboration

Collaborating in partnership with the interprofessional team required mutual trust between parents and health care professionals. Parents reported that a trustful relationship was established when the chemistry was right, when the health care professional invested sufficient time in conversations and addressed the parents’ issues and when the parents were the center of attention of the health care professionals. Parents established especially close and sustainable relationships with the primary care nurse, the midwife and the APN.

##### Perception of Health Care Professionals’ Roles and Competencies

Particularly at hospital right after preterm birth, when many different health care professionals were involved, parents felt challenged. The unfamiliar situation, together with uncertainties about the different scopes and roles of the health care professionals, was demanding for parents. During this phase, many parents focused on the primary care nurses and physicians who were involved in daily care of their preterm infant. Other health care professionals were often disregarded or less accepted by the parents. During the initial phase at hospital, many parents were also unsure about the role of the APN. Some parents experienced the involvement of the APN as a burden. Other parents had the impression that the APN itself was not able to clearly differentiate her role from other professionals.

*“At the beginning, when the role (of the APN) wasn’t fully clear to me, I sometimes wondered why she was also present. There were already so many people.”*

(D26; 26:78)

After discharge, at home, parents were confused when advice on the same topics differed between health care professionals (e.g., the APN, the midwife and the parental counsellor of the family advisory service). However, parents also noted that the collaboration between the APN and other health care professionals worked well when there was a frequent interprofessional exchange. A well-coordinated collaboration made parents feel secure.

*“The APN and the midwife had arranged alternating visits. Thus, someone came to see us at home every week. This was very convenient.”*

(D8; 8:345)

##### The Impact of Different Forms of Communication

The communication of health care professionals with the parents, but also among each other, had a strong impact on the parents’ well-being. The communication form was often criticized, as was a missing or misleading communication.

*“…if they didn’t communicate such things (diagnostic findings). You can’t communicate nothing to a waiting family. Even if one doesn’t say anything, you realize their facial expressions and you know what’s up.”*

(D50; 50:89)

As a result, parents felt insecure. Situations were described as particularly stressful when parents received different or conflicting responses from different professionals. Often the parents then focused on the health care professional they deemed to be the most competent.

One-way communication or sole provision of information without considering the questions or needs of parents resulted in frustration. Parents experienced many professionals who communicated with a focus on disorders of their preterm infant and potential complications. In contrast, the APN, midwife, musical therapist or psychologist communicated positively and were motivating and encouraging.

*“They were always positive, even if something was going on, they always saw something positive. Not like the others, who then said, oh the child is ill, so ill.”*

(D16; 16:44)

##### The Impact of Interprofessional Roundtable Discussions

The intention of the interprofessional roundtable discussions offered within the “Transition to Home” model was to facilitate the collaboration between parents and health care professionals and to define the most appropriate therapeutic and supportive interventions for the family. Generally, parents remarked very differently on these discussions, highlighting various benefits and barriers. Whenever possible, both parents participated in these discussions. If this was not possible, the APN stepped-in and represented the parents. However, parents preferred to be personally involved and thus experienced roundtable discussions in which they were represented by the APN as less productive. Parents expected to contribute significantly to the discussions and were usually well prepared, with the support of the APN. Parents who prepared for the discussions mostly commented positively; parents who were unable to contribute to the discussion or who were not invited to take a more active role considered the discussions less helpful.

According to the parents, the discussions primarily addressed the health status of their child from the point of view of the different health care professionals, assessing the steps and milestones of their child’s development. The needs of the parents were also discussed. A frequent discussion topic was discharge from hospital and the corresponding support needs of the family. Parents regarded the discussion about discharge and follow-up needs as particularly relevant and beneficial. Consequently, parents participated more frequently in the roundtable discussions while the infant was in hospital compared to the period after discharge.

If, during the discussions, any issues or conflicts were resolved and a consensus on the procedures was reached, parents felt relieved. Knowing that all of the professionals around the table cared about their child and were trying to find the most suitable solution was a very positive and reassuring experience. Parents, in fact, expected that the roundtable discussions would welcome different opinions and then focus on working together to identify the optimum solutions.

*“I see it like this: you learn about the problem and then discuss together how to handle it”*

(D50; 50:69)

In contrast, if there were no issues at all, the infant developed as expected and the parents felt sufficiently informed, there was no interest in interprofessional discussions. Parents described the discussion as a waste of resources when the aim of the conversation was unclear or when there were too many health care professionals attending who contributed little to the discussion. Parents benefited from the roundtable discussions depending on the participating health care professionals. The most relevant professionals for parents were the neonatologist, the primary care nurse, the APN and the midwife. If one of these professionals was not attending the discussion, parents questioned its use and, to some extent, felt treated disrespectfully. In a smaller group, parents experienced better discussions, while larger groups could even be perceived as threatening.

*“There were so many people. At first we thought that we massively underestimated the situation. Does it need so many people to support us?”*

(D12; 12:50)

## 4. Discussion

Through the findings of our study, we gained a deeper understanding about how families experienced the support of an APN-led, interprofessional model of care after preterm birth. The interviews with parents revealed three main themes: TtH and the relevance of continuity of care, enhancement of parents’ autonomy and self-confidence and the perception of interprofessional collaboration. These main themes are strongly interconnected to each other and represent key elements of the FCC concept [8]. FCC interventions empower parents and thus contribute to their sense of autonomy; parents gain security, encouragement and are supported in challenging or demanding situations [30].

In our study, an approach based on the principles of the FCC was predominantly provided by the APN. This led to parents feeling secure and autonomous in caring for their premature infants, particularly at home. However, a sustainable enhancement of the parents’ autonomy requires the implementation of an FCC approach hospital-wide and beyond the hospital stay. This requires a paradigm shift within the hospital. FICare is a specific and comprehensive FCC approach which addresses this issue. It aims at integrating parents into the care team during the hospital stay, and it is an option to improve the FCC approach [12]. The FICare approach thus includes training the entire care team in the FCC approach [12].

The continuity of care and having a defined health care professional as an anchor over the entire care continuum was the most relevant aspect from the parents’ perspective. This aspect of continuity lays the foundation for the development of a partnership between the family and health care professionals. On the one hand, the health care professionals continuously obtain more insights into the family, allowing them to better address individual needs [31] and to communicate in an appropriate, family-oriented way [15,30]. On the other hand, parents benefit from the expertise and guidance provided to them.

The parents’ views clearly reflect the value of continuity whenever they described their experiences of unmet expectations in disruptive situations. For health care professionals, such changes are common; for parents, they represent a challenge as they must adapt quickly to a new situation that is unfamiliar to them. This adaptation requires more effort from the already stressed parents. Disruptions lead to frustration and loss of trust [31]. To reduce the associated stress, the management of such disruption plays an important role. Awareness and specific education of health care professionals regarding these aspects is necessary [31,32]. Seppänen et al. [32] highlight the optimized coordination of care and follow-up as well as appropriate communication strategies to overcome disruptive situations.

Communication plays a major role in the entire care continuum and has an impact on parents’ stress levels and self-confidence [15,33]. The parents in this study confirmed that clear, unambiguous and consistent communication made them feel supported, respected and secure. Communication that was perceived as particularly supportive, motivating and consolidating focused on the strengths and resources of the infant and the family. This study thus further highlights the relevance of communication, as previously reported in various studies. Jeukens-Visser et al. [15] described the importance of a strength-based and non-judgmental communication approach, where the attitude of health care professionals plays a central role and parents are seen as experts. Wreesmann et al. [30] denoted communication as the basis to promote parents’ partnership participation in care of their infant, leading to positive care outcomes. They developed a specific communication framework for families with preterm infants [30]. This reflects parents’ views in our study and supports the implementation of FCC.

An exchange of information includes the provision of clear, accurate and timely information but also inviting parents’ knowledge and opinions. Parents must be part of decision-making and must be positively empowered to care independently for their preterm infant at all stages of the transition period. Wreesmann et al. [30] recommended to pay attention to topics and goals that are discussed with parents, as well as to where and how these goals are reviewed. Their communication framework could be a basis to optimize the interprofessional roundtable discussions we introduced in our TtH model. The interprofessional roundtable discussions were aimed at achieving a more family-centered care experience and, in particular, to foster collaboration between parents and health care professionals. Although family-centered rounds are known to provide parents with opportunities to enhance their self-confidence and trust [34], parents in our study mentioned barriers to successful discussions.

Parents will naturally engage in the care of their preterm infant but want to be recognized as a partner [18]. In our study, this was the basis to enhance parents’ knowledge and self-confidence to ensure they were prepared for the transition from hospital to home. Parents who participated in a Swedish post-discharge program highlighted the importance of feeling secure and of being supported by a specialized health care professional from the hospital who guaranteed continuity of care and focused on the resources and strengths of the family [35]. Similarly, in our study, nutritional aspects and the development of the infant were the topics of highest interest to the parents. While knowledge transfer at the hospital was mainly provided by nurses or therapists, this changed after discharge when the midwife and especially the APN were the key professionals who provided parents with knowledge. In this respect, our study showed that the expertise in neonatology of the APN was particularly helpful during the first weeks after discharge, especially when the preterm infant was born at an early gestational age.

Despite its family-centered approach, it appeared that the TtH model did not accommodate the needs of fathers sufficiently well. Although most fathers wanted to make use of the interventions offered and were willing to take responsibility for the health of their child and the mother, they noted similar barriers as reported in other studies, e.g., obligations related to work or caring for other family members [18]. Hemle Jerntorp et al. [36], who evaluated a family-centered care program in the NICU, reported that in this context, fathers often felt excluded and that it focused on family needs rather than their own needs. It is thus important that health care professionals are sensitive towards the needs of fathers and make a particular effort to include them.

In our study, fathers barely made use of the psychological support offered. Given the evidence that fathers can also suffer from emotional effects after preterm birth, such as depression, anxiety or post-traumatic stress disorders, and that this can have a negative impact on their relationship and interaction with the child [4,37], it is important to make the psychological support easily accessible [18]. Using eHealth applications such as telemedicine or video consultations can be an option for a low-threshold service for fathers [38].

Interestingly, it should be noted that during hospitalization in NICU, mothers also barely used the psychological support offered by the TtH model. Most parents did not regard this support as crucial at this time. Using a validated tool to specifically screen for emotional needs of parents is, however, recommended by Hynan et al. [38]. The authors also suggest to regularly perform such a screening after discharge. In our study, the APN did ask the parents about their emotional needs during the home visits but did not perform a systematic screening.

To address the emotional needs of the parents, TtH also included a non-medical element in the form of creative music therapy, which is known to be beneficial in an NICU environment [39]. Such a psychosocial approach empowers parents to communicate with their infants through the subconscious parts of music and physical contact [40]. While the inclusion of such a non-medical element can be regarded as complementary, parents commented favorably on this format in our study. Although music therapy is one of the most often explored alternative therapeutic approaches with a proven positive impact on families with preterm infants, there are several new complementary interventions with potential positive effects on parental mental health: narrative writing, art therapy [41], guided imagery [42] or massage of the preterm infant by parents [43].

Regarding the lead healthcare professionals, different family-centered care models implemented different approaches. A trained nurse was responsible for in-hospital education and home visits after discharge in the modified MITP [16] and a pediatric physiotherapist was in the lead in the TOP Program [15], while the SPIBI program [14] engaged different professionals (e.g., neonatal nurses, psychologists, neonatologists, physiotherapists) who conducted the interventions. In the TtH model, an APN was the key person. As the other models show, different healthcare professionals can ensure continuity of care. However, parents in our study expected a specific expertise regarding preterm infant and competencies in coaching and supporting in complex situations, as well as communication and leadership skills, all of which are defined as being part of an APN role [22,23]. Hence, an APN-role that is linked to neonatology and/or midwifery seems appropriate. From a parents’ perspective, the competencies and capabilities of the APN were not always clear, and parents sometimes had difficulties assigning the relevant responsibilities to the different healthcare professionals, resulting in confusion and uncertainty. The new role implemented in our model and a possibly not yet fully defined scope of practice might have contributed to this. Parents commented that they realized the potential of the APN only during the process. Introducing the APN as early as possible, repeatedly explaining their role in the model and further increasing the visibility of the APN during the in-patient phase could be measures to clarify the competencies of the APN and thus give better guidance and clarity to parents. Furthermore, it must be noted that in countries where the scope of practice is regulated, greater role clarity has been achieved [23]. Switzerland, in contrast, does not yet have a legal framework for APN roles [25]. Improving the legal framework would advance the introduction of novel models of care in the longer term.

Parents in our model felt secure through ensuring continuity of care and well-functioning and coordinated interprofessional collaboration across the continuum of care. Well-coordinated interprofessional collaboration is challenging and requires the health care system to be prepared. In Switzerland, incentives and various initiatives that promote interprofessional collaboration, coordination and integrated care have increasingly emerged over the past few years [44]. Health care systems stakeholders should support and ease these developments by testing innovative person- and family-centered care models and alternative reimbursement models and by providing incentives for interprofessional collaboration. Thus, system thinking by all stakeholders in the health care system is required [44].

After an adaptation of the TtH model, we recommend re-evaluating the program. A re-evaluation could include the introduction of a standardized evaluation scheme, for example, using patient-reported experience measures (PREMs). The measurement of PREMs is aimed at determining the perception of patients’ experiences of a care process, including, for example, satisfaction, subjective or objective experiences and the observation of health care professionals’ behaviors [45]. A standardized evaluation approach would support continuous monitoring, sustainability and further improvement of the TtH model. Before implementing the adapted TtH model in another hospital, it is recommended to analyze the context to detect barriers and facilitators that have to be addressed when implementing such a model of care [46].

### Strengths and Limitations

The methods applied to develop the interview guide and to analyze the results are well-established. The interview number was large enough to reach data saturation. Although we conducted interviews with 39 parents who were part of the TtH program and who followed the entire six-month support period after hospital discharge, we reached data saturation after the analysis of 34 interviews.

The study sample was characterized by an average parental age over 30 years, a high education level and a higher income than the Swiss average. This might have influenced the parents’ needs and expectations toward care after preterm birth [32]. In addition, the inclusion criteria of this study may have led to a certain selection bias. Parents who were not able to speak German, French or English were excluded. Cultural differences and/or previous experiences in the health care system can shape parents’ needs and expectations. Despite the potential limitations, the results showed a clear consistency with respect to the main themes and are thus valuable. Furthermore, the focus on parents as the core stakeholders in such a model of care is regarded as a strength of this study. In particular, the inclusion of fathers is considered important, as their views are less present in published studies. By conducting individual interviews with mothers and fathers separately, this ensured fathers’ specific views and experiences were captured and recorded.

To the best of our knowledge, the “Transition to Home” model is one of the few existing interventions that aims to support parents after preterm birth, along the entire continuum from birth: during NICU stay, over discharge and covering the first months after discharge. The model bridges from hospital to home and, depending on the needs of the family, then hands over to out-patient care services. It must be considered that the “Transition to Home” model was specifically tailored to support families after preterm birth and that it was only introduced in 2018. In addition to providing a more general view on how parents perceived the care, the interviews are therefore also part of the evaluation of this model and will contribute to eventually optimizing the model.

## 5. Conclusions

This study evaluated a model of care related to preterm birth, the “Transition to Home” model, from the perspective of the core stakeholders, i.e., the families involved. Generalizing the key elements that were identified, it can be stated that continuous, family-centered care and interprofessional well-functioning collaboration promote the well-being of the family after a preterm birth. To meet the needs of the family, these aspects must be covered in a corresponding model of care. The unambiguous implementation of an FCC approach by all health care professionals is necessary. Emotional support complemented by non-medical approaches and strength-based and family resource-oriented forms of communication were identified as central aspects.

Discharge of the family from hospital to home represents a significant disruption. If the factors identified in this study are applied, this transition can be smoothened to the benefit of the family. The close guidance of the family has the potential to prevent the escalation of issues through early intervention and can, thus, also have a positive impact on the health care system.

## Figures and Tables

**Table 1:** Main Components of the Model ^1^.

Components of Model	Description
Advanced Practice Nurse (APN) support	All team members contribute to a comprehensive plan for individual discharges, hold consultations, coordinate and collaborate closely with different HCPs so that information flows freely, and they participate in regular interprofessional exchanges. The APN takes a family-centered approach in assessing the needs of the families and in making shared decisions. The APN regularly visits, consults with and educates parents and acts as a continuous partner. After discharge, the APN offers three systematic follow-up calls, telephone support when needed and up to nine follow-up home visits to assess the physical health of infants and parents and the mental health of parents, with a view of evaluating interventions and adapting the care as the family’s needs evolve.
Psychological support	A psychologist provides psychological support to all families, comprising assessment and at least three follow-up consultations before the infant is discharged. The goal is to re-establish emotional stability, improve parents’ ability to cope, prevent the parents and family from developing adaptive disorders and protect the infant from developmental disorders.
Lactation consultation	During hospitalization, the lactation consultant responds to the needs of the families, including fathers. The aim is to strengthen parent–child bonds and to show parents how to meet their child’s nutritional needs.
Physical therapy	The physical therapist provides treatment after an assessment. In a single consultation, the family learns how to handle their premature infant in everyday life, in a manner appropriate to the infant’s developmental stage.
Support by social worker	Social workers collaborate closely with the APN and are involved with every family. They help families cope with daily life after preterm birth and during and after hospitalization.
Music therapy	A music therapist offers music therapy during hospitalization to stabilize the child, support its development, reduce parents’ anxiety and enhance their self-efficacy.
Interprofessional roundtable discussion	Interprofessional roundtable discussions with involved HCPs and parents are held twice while the preterm infant is hospitalized and once three months after discharge. The meetings seek consensus on the optimal support for families in care.

^1^ Table first published in Schuetz Haemmerli et al., 2021 [21].

**Table 2:** Sample Characteristics.

Characteristic	Mothers N = 20	Fathers N = 19	Infants N = 22
	*n* (%) or median (IQ-range) or mean ± sd	*n* (%) or median (IQ-range) or mean ± sd	*n* (%) or median (IQ-range) or mean ± sd
Age, years	32.5 (31.0; 35.8) 33.3 ± 2.8	35.0 (31.0; 38.0) 36.5 ± 8.1	
Nationality			
Swiss	16 (80%)	16 (84%)	
German	1 (5%)	2 (11%)	
Macedonian	1 (5%)		
Italian		1 (5%)	
Other	2 (10%)		
Marital status			
Married	14 (70%)		
Unmarried	6 (30%)		
Living in Switzerland since			
Birth	15 (75%)	16 (84%)	
>20 years	1 (5%)	2 (11%)	
>5 years	2 (10%)		
>2 years	1 (5%)	1 (5%)	
<2 years	1 (5%)		
Highest education level			
Primary and secondary school	2 (10%)		
Apprenticeship	3 (15%)	5 (26%)	
College of higher education	6 (30%)	1 (5%)	
University of applied science	3 (15%)	2 (11%)	
University	6 (30%)	9 (47%)	
Other		2 (11%)	
Employment status			
Full-time	5 (25%)	11 (58%)	
Part-time	11 (55%)	5 (26%)	
Not employed	4 (20%)	3 (16%)	
Yearly family income			
40,000–60,000 Swiss francs	1 (5%)		
60,000–80,000 Swiss francs	3 (15%)		
80,000–100,000 Swiss francs	5 (25%)		
>100,000 Swiss francs	11 (55%)		
Method of delivery			
Planned caesarean	6 (30%)		
Unplanned caesarean	12 (60%)		
Vaginal delivery	2 (10%)		
Multiple birth	2 (10%)		
Infant’s gender			
Male			11 (50%)
Female			11 (50%)
Gestational age at birth, weeks			28.0 (26.0; 32.8) 29.0 ± 3.3
Birth weight, g			1097.5 (706.3; 1677.5) 1209.5 ± 548.3
Birth length, cm			40.0 (32.8; 43.5) 38.6 ± 5.8
Length of hospital stay, days			63.0 (28.3; 94.8) 71.7 ± 52.9

**Table 3:** Results of the thematic analysis of the parent interviews.

Thematic Analysis of the Parent Interviews			
**Main theme**	TtH and the relevance of continuity of care	Enhancement of parents’ autonomy and self-confidence	Perception of interprofessional collaboration
**Sub-themes**	The APN: the bridge home Continuity of care and parents’ unmet expectations	Acquiring knowledge Parental emotional support	Perception of health care professionals’ roles and competencies The impact of different forms of communication The interprofessional roundtable discussions

## Data Availability

The data presented in this study are available on request from the corresponding author. The data are not publicly available due to privacy and ethical reasons.

## Supplementary Materials

The following are available online at https://www.mdpi.com/article/10.3390/ijerph19074309/s1, Table S1: “Transition to Home” Interviews with parents 6 months after discharge.
