# Supplementary material for: Enhancing Parents’ Well-Being after Preterm Birth—A Qualitative Evaluation of the “Transition to Home” Model of Care
Source: Int J Environ Res Public Health. 2022 Apr 4;19(7):4309. doi: 10.3390/ijerph19074309 (PMC8998674; doi:10.3390/ijerph19074309)
Supplement: Supplementary file 1 [file ijerph-19-04309-s001.zip › ijerph-1645094-supplementary.pdf]

International Journal of
Environmental Research and Public Health

MDPI

Supplementary Materials – Interview Guide

Table S1. "Transition to Home" - Interviews with parents 6 months after discharge

**Introduction and General Questions**
• How are you? How is your child?<br>• Mr/Ms. X, your child/ren (name/names of children) was/were hospitalized at the neonatology unit and you were included in the "Transition to Home" program. We conduct this interview to learn more about your view regarding this program. How did you experience the overall care and support?<br>• Which moments were especially nice /impressive? Which moments were difficult/stressful?<br>• How did you experience the collaboration with the staff/ professionals involved?
**Experiences with the Advanced Practice Nurse and the support within the Transition to Home model**
**During the last weeks you have received support from the Advanced Practice Nurse.**<br>• How did you experience the first contact with the APN and the support obtained during your stay at hospital?<br>• What was especially positive and helpful? What was difficult?<br>• Which additional support would you have needed?<br>• How did you experience the discharge from hospital?<br>• How well prepared did you feel for the discharge?<br>• What was especially helpful and supporting in this moment?<br>• What did you miss concerning the transition to home and the time after that?<br>• How did you feel at the day of discharge?<br>• How did you feel on the first day at home with your child?<br>• How did you experience the support provided by the APN at home?<br>• What was especially positive and helpful? What was difficult?<br>• Which additional support would you have needed at home?<br>• How did you experience the coordination and interaction of the APN and the other services after discharge from the hospital?<br>• How did you experience the collaboration with the APN?
**Experiences with interventions from different health care professionals**
**As part of the TtH model you had contact with various health care professionals. You have experienced different ways of support and forms of therapy.**

- From which health care professionals did you receive support?
- What was helpful and positive? What was less helpful?
- Which additional support would you have needed? What did you miss?

We are interested in how you experienced the different professional interventions.

[If appropriate, asking the following set of questions with respect to psychological support/ lactation counselling / physiotherapy/ social counselling / musical therapy/ round table discussions]:

- How did you experience the support by the psychologist/…/…?
- What effect had this psychological/…/… support on you as a mother/father?
- Which effect had the psychological care/…/… on the relationship between you and your child?
- What did help you to cope with the situation? What was difficult for you? What did you miss?

Satisfaction with the model

Looking back at your overall experience with "Transition to Home" …

- How satisfied are you with the support in general?
- What was especially positive/helpful? What was less helpful?
- What would you change in the model of care?
- As how helpful do you consider the model for other parents?

Study burden

The participation in the pilot study was an extra effort for you…

- How did you experience the extra effort caused through the study participation?
- How would you decide about the participation today?

Final Questions

- How would you describe your current parental self-confidence?
- How do you feel regarding the near future?
- Is there something you want to tell me that I didn't ask for?
- Do you have any recommendations for us for the future care of preterm infants and their parents?
